# Reasons for leaving home and pattern of child abuse and substance misuse among street children in Khartoum, Sudan: a cross-sectional survey

**DOI:** 10.11604/pamj.2023.46.36.33887

**Published:** 2023-09-25

**Authors:** Suha Mohammed Elhassan Ali Hassan, Satti Abdelrahim Satti, Mohammed Abdulrahman Alhassan

**Affiliations:** 1 Gaafar Ibn Auf Pediatric Tertiary Hospital, Khartoum, Sudan,; 2 Department of Pediatrics, Faculty of Medicine, Almughtaribeen University, Khartoum, Sudan,; 3 Department of Pediatrics, College of Medicine, Prince Sattam Bin Abdulaziz University, Al-Kharj, Kingdom of Saudi Arabia

**Keywords:** Homeless youth, child abuse, drug users, Sudan

## Abstract

Street children are particularly susceptible to health-related adversities, including those resulting from substance abuse and child abuse. Information on street children is deficient in Sudan. This study provides basic data on characteristics, factors for leaving home, the pattern of child abuse and substance misuse among street children in Khartoum State, Sudan. This is a descriptive, cross-sectional, and community-based study. Data were collected through direct questioning of a sample of street children using a structured, standardized, and pretested interview-administered questionnaire. Two hundred and seventy-five (275) street children were interviewed. Most street children were males (83%). Of the interviewed children, 36.7% were illiterate, 66.1% had a single parent, and 36% did not recognize a home to return to. The commonest reported reasons for being on the streets were family conflicts and financial/economic difficulties (28.4 % and 27.5%), respectively. 89.1% of the children admitted to being substance abusers, mostly of glue (86.5%) and smoked tobacco (67.3%). Seventy-five-point three percent 75.3% of the children reported being subjected to a form of abuse, with physical and sexual abuse reported by 70.2% and 27%, respectively. Of the 74 children who reported sexual abuse, 49 were males (29% of males), and 25 were females (65% of females). The survey results are thought to guide further research and shape appropriate policymaking and coordinated interventions by concerned stakeholders, whether governmental or non-governmental.

## Introduction

Good child health and well-being are pivotal to sustainable development [1] besides being fundamental rights of every child [2]. Street children are particularly susceptible to health-related adversities. The term “street children” has been defined by the United Nation's Commission on Human Rights as “any girl or boy (aged under 18 years) for whom the street has become his or her habitual abode and/or source of livelihood, and who is inadequately protected, supervised or directed by responsible adults” [3]. “Street children” is a widely witnessed phenomenon worldwide, with increasing numbers mainly in developing countries. In Sudan, the problem is still worsening and is exacerbated by the economic difficulties and security disturbances going through some parts of the country. The separation of South Sudan, internal displacement due to poverty, desertification, and conflicts, and the limited resources to deal with the issue have notably deepened the problem [4].

Street children are often exploited and exposed to a wide range of abusive victimization, threatening their health and well-being. Being on the streets most of the time makes them particularly more vulnerable. These adversities frequently lead to serious physical and mental health issues or even premature death [5]. The increased propensity towards antisocial and violent behavior has been well documented among street children [6]. Abandoned children form gangs, create their own argots, and engage in petty thefts, prostitution, and commercial sexual exploitation.

Information on street children is deficient in Sudan. Through conducting this study, we intended to find answers to the following questions: what are the factors that lead them to leave home and/or live on the streets? What are the types and patterns of abusive assaults they face? What are the types and patterns of substance abuse they undertake? This study aims to collect basic data on these issues by directly questioning a sample of street children in Khartoum State, Sudan. We believe these baseline data will be invaluable in informing community measures and relevant policy decision-making in the attempt to address the root issues pertinent to the phenomenon of street children in Sudan.

## Methods

**Study design and setting:** this is a descriptive, cross-sectional, and community-based study carried out in Khartoum State, the capital of Sudan. It included those children found on the streets and homeless ones found in temporary shelters and gathering points established by non-governmental organizations. Data were collected from November 2019 to August 2020.

**Participants, variables, data source, and study size:** we used the term “street children” to refer to the population of children who live on the streets and those found in temporary non-governmental shelters. Street children between 5 and 18 years of age, who are mentally normal and who have no communication problems, were included. Data were collected using a structured, standardized, and pretested interview-administered questionnaire. In addition to initial demographic data, the questionnaire contained closed-type direct questions, focusing on three main areas: the reason for leaving home, exposure to different types of abuse, and substance abuse. Children were interviewed individually after obtaining verbal consents. At the interview, definitions and explanations of technical terms (e.g., abuse) were provided for children where needed. The World Health Organization (WHO) Consultation on Child Abuse Prevention definition of child abuse and the WHO Expert Committee on Drug Dependence definition of substance abuse were adopted in this study [7,8]. The sample (275 children) was selected purposively from gathering places, main street intersections, as well as makeshift gathering sites of non-governmental organizations where food, health care, and social activities were provided on the go. The target locations were agreed upon a priori and new locations were added by asking the interviewed children. We attempted to interview as many children (who consented to participate) as possible within the target range and inclusion criteria. The first author conducted all the interviews and collected the data.

**Statistical methods:** the data were analyzed using the statistical package of social sciences (SPSS) version 20.0. Descriptive statistics were used to summarize the data on variables related to reasons for leaving home, patterns of abusive assaults, and substance abuse and were presented as frequencies and percentages. Bivariate analysis was used to examine variables associated with substance misuse and being a subject of abuse. This was done using a chi-square test. A p-value of 0.05 or less indicated statistical significance.

**Ethical considerations:** written permission to undertake the study was obtained from the Ministry of Social Welfare and Shelter. Verbal consent was obtained from each child.

## Results

**Participants’ characteristics:** two hundred seventy-five street children were interviewed. Males were 229 (83%), and females were 46 (17%), with a male-to-female ratio of 5 to 1. Their ages ranged from 6 to 18 years (Table 1). Most children (81.1%) were Sudanese; the rest were from South Sudan, Chad, and Ethiopia. A third of those from inside Sudan were originally residents of Khartoum State. More than a third (36.7%) of children were illiterate, while 63.3% received some form of education, with one child reaching secondary school. Most interviewed children (66.1%) reported loss or absence of one or both parents at home, mainly due to death, then divorce or separation (Table 1). The time they spent on the streets ranged between a minimum of days and a maximum of 16 years. A quarter (26.6%) of children had one or more siblings or family members accompanying them on the streets, while the rest (72.4%) were on their own.

**Table 1:** characteristics of interviewed children

Characteristic	no. (%)
**Gender**	
Male	229 (83.3 %)
Female	46 (16.7 %)
**Age in years (range; mean)**	6 - 18; 14
**Country/place of origin**	
Sudan (Khartoum)	92 (33.5 %)
Sudan (other locations)	131 (47.6 %)
South Sudan	44 (16.0 %)
Chad	7 (2.5 %)
Ethiopia	1 (0.4 %)
**Highest education reached**	
Illiterate	101 (36.7%)
Khalwa (traditional Quran preschool)	35 (12.7 %)
Basic school	138 (50.2 %)
Secondary school	1 (0.4 %)
**Duration of being on streets in months (range; mean)**	1.8 - 192; 37
**Primary factor for leaving home**	
Family conflicts	101 (28.4 %)
Economic/financial difficulties	98 (27.5 %)
Exposure to abuse	77 (21.6 %)
Abandonment	45 (12.6 %)
Others	35 (9.8 %)
**Parents´ status**	
Absent one or both parents	182 (66.2%)
Both parents present	93 (33.8)
**Causes of absent parent(s); n= 182**	
Death	122 (67.0%)
Divorce or separation	60 (33.0%)
**Previous or current substance abuse**	
Yes	245 (89.1 %)
No	30 (10.9 %)
Substance misused*	
Glue (tire rubber glue solution) sniffing	212 (86.5 %)
Tobacco smoking	165 (67.3 %)
Used snuff	55 (22.4 %)
Locally made alcohol	37 (15.1 %)
Cannabis and related substances	14 (5.7 %)
Medicated cough remedies	16 (6.5 %)
Others	2 (0.8 %)
**Acknowledgment of being subjected to abuse**	
Yes	207 (75.3 %)
No	68 (24.7 %)
Types of abuse reported*	
Physical	193 (93.2 %)
Sexual	74 (27 %)
Emotional/psychological	90 (43.5 %)

* Multiple choice allowed

**Reasons for leaving home and/or living in the streets:** regarding home availability, 31.6% of children reported having homes where they returned to regularly, 32.4% had homes but never got back to them, and 36% did not know a home to return to. The commonest reported reasons for leaving home/ being on the streets were family conflicts and financial/economic difficulties (28.4 % and 27.5%), respectively, followed by abuse and abandonment (Table 1). Activities they reported to practice were begging for money and food (78.5%) and light works (67.3%); 1.5% admitted being involved in street gangs and having committed some form of offense.

**Substance abuse:** two-hundred-forty-five (89.1%) of the interviewed children admitted to being substance abusers, mostly glue-sniffing (bike tire glue solution) (86.5%) and smoked tobacco (67.3%). However, 14 (5.7%) children have admitted to misuse cannabis or related substance (Table 1). There was a statistically significant (p< 0.05) association between home availability and drug abuse; it was also found that the more time they spent on the streets, the more the child is liable to be a drug abuser.

**Physical and sexual abuse:** two-hundred and seven (75.3%) children reported being subjected to a form of abuse, with sexual abuse reported by 43.5%, while 24.7% denied any abuse (Table 1). Of a total of 193 children who were abused physically, it was found that 163 were males (96.4% of males), 30 were females (79% of females). Of the 74 children who reported sexual abuse, 49 were males (29% of males), and 25 were females (65% of females). Higher rates of abuse were among those aged more than 12 years. Children who reported being subjected to abuse of any kind were significantly more likely to be substance abusers and to have spent more time homeless. Those who had homes and regularly returned to, were the least to report any type of abuse. Half of the street children (58.5%) did not notify another individual or authority about being abused, while 7.2% reported informing local police.

## Discussion

The remarkable five-fold ratio of homeless boys to girls could be attributable to socio-cultural and biological considerations. The typical gender role that portrays males as more exposed to and capable of adapting to street life and their natural inclination to risk-taking and peer-motivated experimentation with substances may play a major role. Females tend to assume more indoor/household roles, including chores and care for younger siblings [9]. Street life is perceived as being more dangerous for girls.

High levels of poor education and school drop-out among street children, as shown in our results, are thought to have a complex relationship with homelessness. Poverty, dysfunctional family dynamics, and poor parental education may contribute to both. However, with the noticeable lack of sensible governmental policies towards the education of homeless children, homelessness becomes the primary factor for terminating education among street children. On the other hand, a lack of education would practically mean a lack of future opportunities for these children. This may create a situation of a vicious cycle in which homelessness leads to poor education, which could in turn reduce the future chances of leaving the street and incorporation into the community.

Single-parenthood is a well-known risk factor for homelessness [10]. The absence of one or both parents in this study was mainly due to death or divorce. This contrasts with the situation in other communities, where the unmarried single mother takes the highest proportion [11]. Reasons for leaving home were comparable to those in the study conducted in Egypt by Salem *et al*. analyzing the sociodemographic characteristics of street children in Alexandria, which confirmed that most street children come from problematic family backgrounds and usually are victims of family breakdown and other sexual and physical abuses at home or in the surroundings [12]. Comparable factors were revealed in Ethiopia where causes forcing “children to run away were ranging from escaping abusive parental punishment followed by poverty, hate of stepparents to parental alcoholic behavior” [13]. Self-reported sexual abuse in this study has again shown similar levels compared to other studies where 26% [14] and 20.9% [15] were reported.

Probably, the most alarming finding in this study is the remarkably high percentage of reported drug use and child abuse. The findings in this study that showed fewer risks of child abuse and substance misuse in children who maintain some connection with a home highlight that family support of any kind and any extent may be significantly helpful. In addition to the established and intuitively deducible factors for the high rates of drug use among street children, there is a high probability that substance misuse is a coping mechanism these children use to maneuver hunger and the physical and emotional pain they face on a daily basis. The higher percentage and different pattern in substance use compared to similar studies may reflect availability [16] but should also necessitate further search into other factors.

A focus group discussion and a structured clinical and psychological assessment would have provided a deeper and broader insight into the many sensitive issues in this study. In fact, each of these issues deserves separate research into their magnitude. For instance, the severity, type, frequency, and health consequences of physical and sexual abuse indeed call for more in-depth scrutiny. Because of the Covid-19 pandemic, the data collection process was restricted, and some areas were difficult to reach due to safety issues.

## Conclusion

The results of this survey provide preliminary data into the problem of street children in Sudan. This is thought to guide further research and hence shape appropriate policymaking and coordinated interventions by concerned stakeholders, governmental or non-governmental. Establishing rehabilitation institutions with appropriate professional staffing represents an immediate necessity, as does reunion/incorporation with original foster families.

### What is known about this topic

- *"Street children" is a widely witnessed phenomenon worldwide, with increasing numbers mainly in developing countries;*
- *Street children are particularly susceptible to health-related adversities, including those resulting from substance abuse and child abuse*.

### What this study adds

- *Since information on street children is deficient in Sudan, this study provides basic data on characteristics, factors for leaving home, the pattern of child abuse and substance misuse among street children in Khartoum State, Sudan;*
- *The commonest reported reasons for being on the streets were family conflicts and financial/economic difficulties; through self-reporting, 89.1% of the children admitted to being substance abusers and 75.3% to being subject of one or more forms of abuse*.
